# Supplementary figures and images for: Incidence and Characteristics of Intraocular Lens Dislocation after Phacoemulsification: An Eight-Year, Nationwide, Population-Based Study
Source: J Clin Med. 2021 Aug 26;10(17):3830. doi: 10.3390/jcm10173830 (PMC8432084; doi:10.3390/jcm10173830)

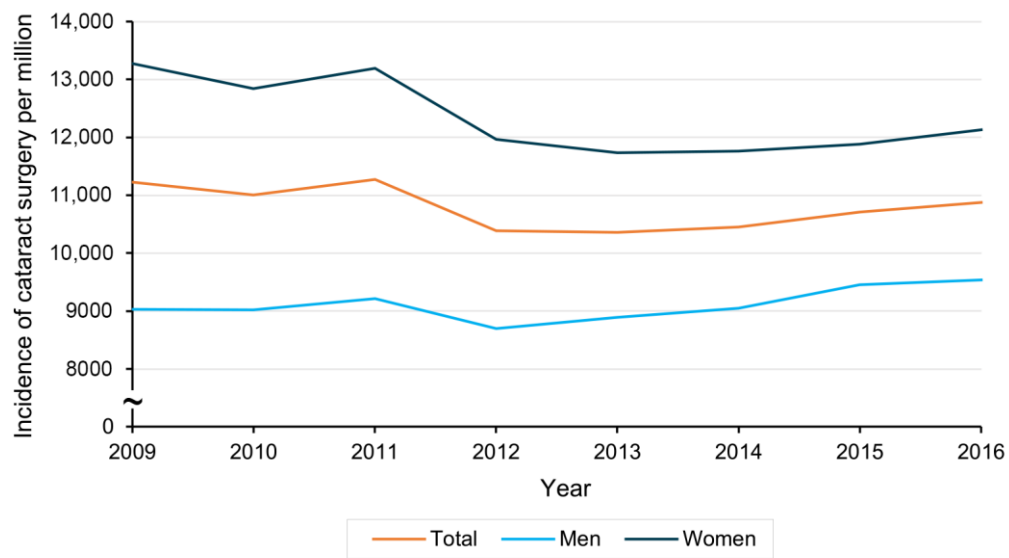

Figure S1: The incidence rate of cataract surgery between 2009 and 2016.

Supplement: Supplementary file 1 [file jcm-10-03830-s001.zip › jcm-1330533-supplementary.pdf]
